# A Treatise on Diseases of the Skin

**Published:** 1895-03

**Authors:** 


*A Treatise on Diseases of the Skin.* By T. M'CALL ANDERSON, M.D. Second Edition. Pp. xx., 761. London: CHARLES GRIFFIN AND COMPANY, LIMITED. 1894.

We are very glad to see a second edition of this work; and when we notice that it comprises an analysis of 12,000 cases which, either in hospital or private practice, have come under the author's personal observation, we are naturally inclined to see what proportions some of the more common skin affections bear to the whole number. We are not surprised that out of the 724 pages in the body of the work, 138 are given to the varieties of eczema and its treatment, 37 to psoriasis and lepra, and 30 to syphilitic affections. Eczema constitutes more than one-fourth of the 10,000 hospital cases, and 34.8 per cent. of the private ones. And whilst we have always understood that scabies was very prevalent in Scotland, we were scarcely prepared to find that the cases in hospital practice numbered precisely the same as those of eczema; or in other words, out of every 100 skin patients seen, a fraction over 50 would be divided equally between eczema and scabies. The practical issue of this is, that the treatment and description of these affections are very full and very satisfactory, and are well worth the attention of the busy practitioner, who will be repaid by some very elegant and practical formulæ. We like the contrasted tables of diagnosis: speaking broadly, they are good and graphic.

In almost every new work on Skin Diseases, or new editions of standard works, we look to see what is said about preparations of tar. These are often *very* beneficial, when they do good at all; but on the other hand, they so often set up severe irritation and annoy patients, that we never prescribe the remedy at first without giving the patient a warning. We are desirous of finding whether any trustworthy guiding principles have been discovered, and hitherto we have not been happy enough to find any. Nor does Dr. Anderson help us in this matter.

Then to glance at a few rarer affections. On page 101, erythema circumscriptum [lichen circinatus (Wilson), seborrhœa corporis (Duhring), seborrhœa papulosa (Crocker)] is described as non-contagious. Dr. Anderson says its appearance is suggestive of parasitic origin. It is certainly auto-infectious. One sentence strikes us as remarkable: "It is a relapsing affection, and in some persons only disappears in summer to re-appear at each succeeding winter." Is not the explanation of this, that flannels are left off in summer to be put on again in the cold weather? Let Dr. Anderson have these flannels thoroughly disinfected by hot stoving, and the patient treated by sulphur ointment or some other parasitic destroyer, and we think the relapses will be much diminished.

The author very mildly admits that boils may be occasioned by micrococci. With treatment which thoroughly emphasises this origin, we very rarely meet with recurrences, or "fresh crops."

For some of the contributions Dr. Anderson is indebted to those who have had very special opportunities of considering the subjects with which they deal.

We have rarely seen a work more carefully revised and corrected, of which no better proof can be given than that, with the exception of "utricatus" for "urticatus" in the index, which is very copious, and perhaps one or two more such trivial errors, there seems no misprint in the work. Upon the whole it is a grand and practical work, and well worthy of the Professor of Clinical Medicine in the University of Glasgow.

